# Berberine Inhibits Eczematous Skin *S. aureus*-Induced Mast Cell/Basophil Activation and Modulates MAPK-Associated Gene Expression

**DOI:** 10.3390/ijms27177875

**Published:** 2026-09-03

**Authors:** Anish R. Maskey, Daniel Kopulos, Madison Spears, Zhen-Zhen Wang, Xian Mo, Ibrahim Musa, Nan Yang, Anna Nowak-Wegrzyn, Danna Chung, Anne L. Maitland, Julie Wang, Raj K. Tiwari, Hugh A. Sampson, Jan Geliebter, Xiu-Min Li

**Affiliations:** 1 Department of Microbiology & Immunology, Touro College of Osteopathic Medicine, Great Falls, MT 59405, USA; amaskey@touro.edu; 2 Department of Pathology, Microbiology & Immunology, New York Medical College, Valhalla, NY 10595, USA; dkopulos@student.touro.edu (D.K.); mspears@student.touro.edu (M.S.); jane.wangzz@outlook.com (Z.-Z.W.); xianmo@gird.cn (X.M.); imusa@nymc.edu (I.M.); raj_tiwari@nymc.edu (R.K.T.); 3 Academy of Chinese Medical Science, Henan University of Chinese Medicine, Zhengzhou 450046, China; 4 Department of Allergy and Clinical Immunology, Guangzhou Institute of Respiratory Health, Guangzhou 510120, China; 5 Department of R&D, General Nutraceutical Technology, Elmsford, NY 10523, USA; 6 Department of Pediatrics, Hassenfeld Children’s Hospital, NYU Grossman School of Medicine, New York, NY 10012, USA; anna.nowak-wegrzyn@nyulangone.org; 7 Department of Pediatrics, Gastroenterology and Nutrition, Collegium Medicum, University of Warmia and Mazury, 10-561 Olsztyn, Poland; 8 Center for Integrative Health & Acupuncture, PC, Mamaroneck, NY 105443, USA; dannachung@gmail.com; 9 Division of Allergy & Clinical Immunology, Icahn School of Medicine at Mount Sinai, New York, NY 10029, USA; 10 Department of Pediatrics, Icahn School of Medicine at Mount Sinai, New York, NY 10029, USA; julie.wang@mssm.edu (J.W.);; 11 Department of Otorhinolaryngology, New York Medical College, Valhalla, NY 10595, USA; 12 Department of Dermatology, New York Medical College, Valhalla, NY 10595, USA

**Keywords:** mast cells, basophils, berberine, MAPK, *Staphylococcus aureus*, eczema, atopic dermatitis, inflammation

## Abstract

*Staphylococcus aureus* (*S. aureus*), a primary exacerbating factor in eczema, remains a prevalent pathogenic public-health threat. The mechanism of mast cell/basophil degranulation in the context of *S. aureus*-exacerbated eczema remains unclear. We sought to investigate the direct effect of mast cell/basophil activation by *S. aureus* isolated from patients with severe eczema undergoing topical steroid withdrawal (TSW) and understand the mechanism of berberine (BBR) in inhibiting this activation. Clinical *S. aureus* strains (N = 8) were isolated from skin swabs of severe eczema patients and confirmed by sequencing. Human basophils (KU812), rat basophils (RBL-2H3) and murine mast cells (MC/9) were pre-treated with BBR for 48 h. and stimulated with heat-killed standard *S. aureus*- and clinical strains for 45 min, and degranulation was measured. BBR’s molecular-targets on basophils were identified by computational modeling and validated by qRT-PCR. BBR prevented degranulation following standard *S. aureus* stimulation in KU812, RBL-2H3 and MC/9 cells. BBR dose-dependently inhibited degranulation in KU812. BBR inhibited TNF-α and IL-4 release, and markedly suppressed the expression of FcεR1 (α, β, γ), TNFα, MAPK1, MAPK3, AKT2, CASP9, and CCND1 following standard *S. aureus* stimulation. BBR dose-dependently inhibited KU812 cell degranulation, markedly reduced TNF-α, IL-4, and MAPK1 and enhanced NFκB1A expression following clinical *S. aureus* strain stimulation. BBR inhibition of *S. aureus*-induced KU812 activation was at least associated with inhibition of MAPK-associated gene expression. This study provides novel insights into BBR’s effect in preventing *S. aureus* and human basophil interaction.

## 1. Introduction

Atopic dermatitis (AD) is a chronic inflammatory skin disease affecting 15% to 30% of children and 5% of adults [1]. It is also referred to as eczema characterized by dry, itchy and inflamed skin. The pathogenesis of AD is not fully understood; however, mast cells and basophils contribute to IgE-mediated allergic diseases, including AD [2]. Mast cell activation is associated with allergic diseases like allergic rhinitis, asthma, and AD [3]. The classical mechanism of mast and basophil degranulation involves the production of allergen-specific IgE following allergen exposure. IgE molecules bind to their high-affinity receptor (FcɛRI) on the surface of mast cells or basophils. Upon subsequent allergen exposure, cross-linking of FcεRI-bound IgE by multivalent allergens triggers intracellular signaling cascades, leading to the secretion of granules containing inflammatory mediators, including prostaglandins, interleukins and pro-inflammatory cytokines [4,5]. Various factors, including infections, stress, certain foods, pseudo-allergens, hormones, and neuropeptides, can prime mast cells and basophils for activation [6]. In AD, mast cells/basophils play a significant role as effectors in recruiting other inflammatory cells [7]. This infiltration into itchy skin lesions intensifies pruritus symptoms by increasing contact between nerve fibers [8,9]. The skin of patients with AD shows significant anomalies in microbial communities when compared to the microbiota of healthy subjects [10]. It has been well characterized that *S. aureus* dominantly colonizes the skin of AD patients. Studies have shown 60–100% *S. aureus* colonization in AD patients compared to 5–30% colonization in healthy patients [11,12,13]. This induced itch is associated with a significant increase in *Staphylococcus* species, and a decrease in beneficial genera such as *Cutibacterium*, *Streptococcus*, *Acinetobacter*, *Corynebacterium*, and *Prevotella* [14,15]. *S. aureus* can trigger rapid and robust MC degranulation, driven by its δ-toxin, which serves as a degranulation-inducing factor [16]. It is believed that δ toxin directly binds to MCs and induces their degranulation, triggering the release of inflammatory mediators such as histamine, β-hexosaminidase, and cytokines, including IL-4 and TNF-α [17,18]. This process amplifies local inflammation, promotes immune cell recruitment, and disrupts the skin barrier, perpetuating the itch-scratch cycle characteristic of eczema. However, it still remains poorly understood how *S. aureus* coordinates basophil degranulation in AD.

The PI3K/AKT pathway orchestrates cellular responses such as activation, survival, proliferation, and mast cell/basophil degranulation. PI3K pathways are attractive targets for the treatment of mast cell/basophil-induced inflammatory disease like AD. In human mast cells/basophils, this pathway is initiated primarily through the activation of the FcεRI receptor and its subunits FcεRIα, FcεRIβ, and FcεRIγ [19]. Upon engagement of the *S. aureus* toxin with IgE bound to the high-affinity FcεRI receptor, phosphoinositide 3-kinase (PI3K) is activated. This also induces the production of pro-inflammatory cytokines, including IL-4 and TNF-α, through the activation of additional signaling intermediates like MAP kinases (MAPK1 and MAPK3) [18,20,21]. These cytokines amplify allergic inflammation and recruit other immune cells to the site of activation. Similarly, AKT2 is an important isoform regulating cell survival and metabolic functions in mast cells/basophils [22]. The regulatory proteins such as NFκB inhibitor alpha (NFkB1A) serve as a check on these pathways, limiting excessive activation and promoting apoptosis and cell cycle arrest [23].

The most widely used treatment approach for mast cell/basophil-associated disease includes the use of drugs such as antihistamines, antileukotrienes, and steroids [24]. Though these treatments provide short-term benefits, the use and overuse of antihistamines and steroids can lead to short-term side effects like nausea, drowsiness, dry mouth, weight gain, mood changes, and long-term side effects like steroid withdrawal, osteoporosis, skin thinning, and increased infection risk [3,25]. Therefore, natural compounds have drawn significant attention as therapeutic agents for various diseases. A compound of interest, berberine (BBR), is an isoquinoline alkaloid, isolated from the rhizomes of *Coptis chinensis*, *Phellodendron japonicum*, and *Berberis aquifolium.* BBR is a multifaceted therapeutic agent, capable of exerting anti-inflammatory, anti-microbial, anti-tumor, and antidiabetic effects [3]. The BBR-containing formula, FAHF-2, has shown persistent protection against peanut anaphylaxis [26]. BBR-containing topical therapy has been shown to improve sleep disturbance, skin lesions, and skin integrity and reduce peripheral blood eosinophil percentage [27]. Furthermore, we showed that BBR inhibits growth of clinical *S. aureus* strains isolated from AD patients [28]. *S. aureus* induces a pro-inflammatory response in AD via mast cell/basophil activation; understanding how BBR counters the effects of these responses is essential [16,28]. Therefore, this study aims to decipher mechanisms associated with BBR inhibition of mast cell/basophil degranulation following stimulation with *S. aureus* isolated from AD patients, providing a potential pathway for targeted intervention in managing AD-associated inflammation.

## 2. Results

### 2.1. BBR Inhibited Standard S. aureus-Induced Mast Cell/Basophil Degranulation

**Figure 1:**
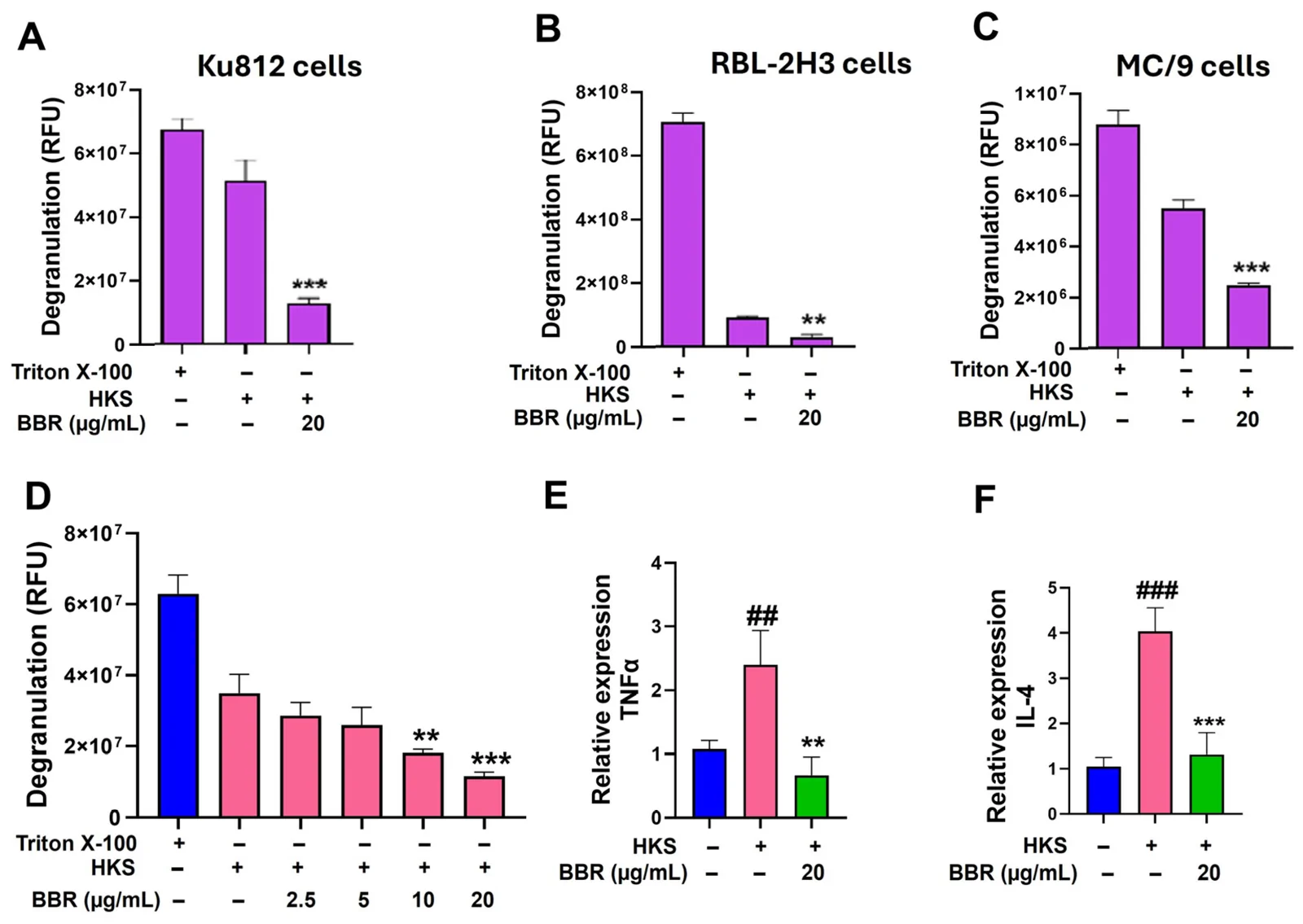
BBR prevented mast cell/basophil degranulation: Human basophil cells (KU812), rat basophils (RBL) and murine mast cells (MC/9) were pretreated with BBR (20 μg/mL) for 48 h., after which cells were stimulated with standard heat-killed *S. aureus* (ATCC #33591) for 45 min. β-hexosaminidase release was measured by DCFDA assay. BBR pretreatment showed inhibition of beta-hexosaminidase release in (**A**) KU812 cells. (**B**) RBL-2H3 cells, and (**C**) MC/9 cells. BBR treatment dose-dependently inhibited degranulation (**D**), and reduced expression of TNF-α (**E**) and IL-4 (**F**) in Ku812 cells following standard *S. aureus* (ATCC #33591) stimulation. Blue—unstimulated/untreated; pink—stimulated/untreated; green—stimulated/treated; (N = 3 biological replicates. ^###^ *p* < 0.001; ^##^ *p* < 0.01 vs. untreated; *** *p* < 0.001; ** *p* < 0.01; vs. HKS; statistical significance was determined by one-way ANOVA). RFU-Relative fluorescence unit. Expression was normalized using GAPDH as a housekeeping gene.

KU812, RBL-2H3 cells, and MC/9 cells were pre-treated with BBR for 48 h, after which they were stimulated with standard *S. aureus.* BBR significantly reduced degranulation as evident by decreased β-hexosaminidase release in all three cell lines (*p* < 0.001) (Figure 1A–C). These results show that BBR has a potent anti-mast cell/basophil degranulation effect. Since the most potent effect was seen in KU812 cells, we used KU812 for our next set of experiments. We further showed a dose-dependent inhibition of β-hexosaminidase release with BBR treatment (2.5, 5, 10, 20 µg/mL) following standard *S. aureus* stimulation (*p* < 0.01–0.001) (Figure 1D). In addition, BBR showed remarkable inhibition of TNF-α (*p* < 0.01) and IL-4 (*p* < 0.001) expression in standard *S. aureus*-stimulated KU812 cells (Figure 1E,F). TNF-α is a key cytokine released following mast cell degranulation and is an important inflammatory cytokine that causes mast cell-mediated detrimental tissue damage [29].

### 2.2. BBR Inhibited Standard S. aureus-Induced Expression of FcεR1 (α, β, γ)

**Figure 2:**
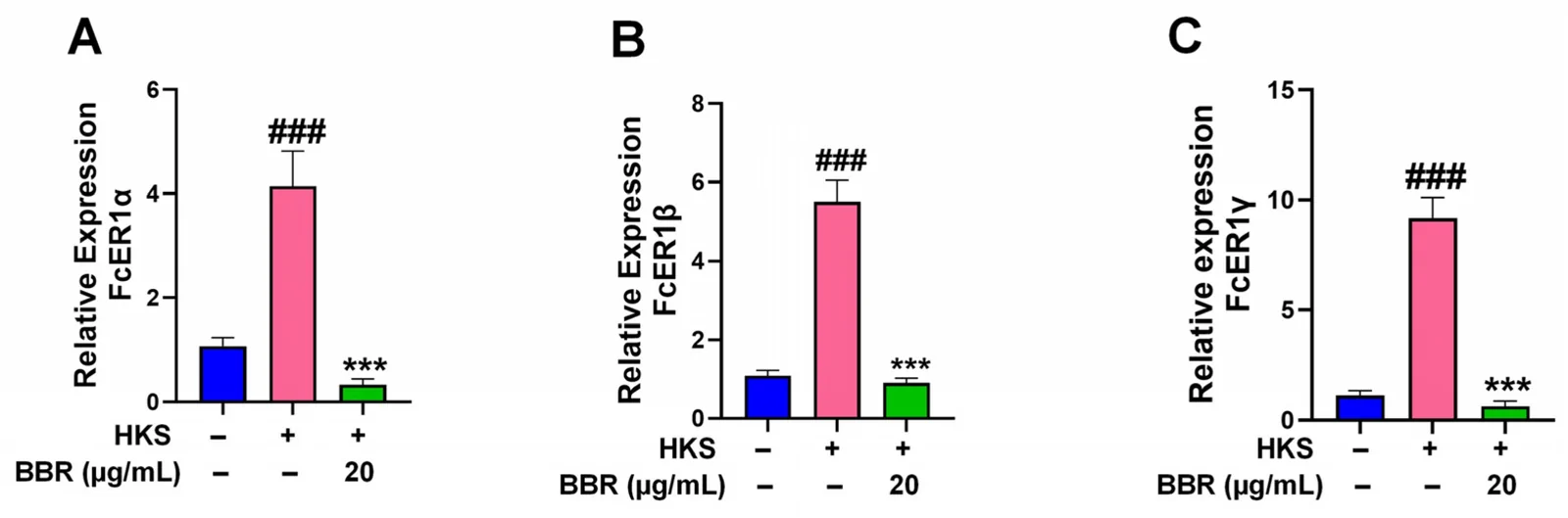
BBR inhibited expression of FcεRI. Ku812 cells were cultured in presence of BBR (20 μg/mL) for 48 h. and stimulated with standard heat-killed *S. aureus* (ATCC #33591) for 4 h. Cells were harvested and gene expression analysis was performed by qRT-PCR. Expression levels of (**A**) FcεRIα, (**B**) FcεRIβ, and (**C**) FcεRIγ. Blue—unstimulated/untreated; pink—stimulated/untreated; green—stimulated/treated; (N = 3 biological replicates. ^###^ *p* < 0.001 vs. untreated; *** *p* < 0.001 vs. HKS; statistical significance was determined by one-way ANOVA).

As BBR inhibited cytokine expression—TNF-α and IL-4—following standard *S. aureus* stimulation in KU812 cells, we studied the effect of BBR on expression levels of FcεR1(α, β, γ). We found a remarkable increase in expression of FcεR1 (α, β, γ) following standard *S. aureus* stimulation. Previously, TLR3-activated mast cells have been shown to up-regulate a number of costimulatory molecules [30]. In this study, BBR markedly inhibited the expression of high-affinity receptor FcεR1 (α, β, γ) (*p* < 0.001) in human basophils (Figure 2A–C). Overall, these results indicate *S. aureus* can induce expression of the FcεR1 receptor and BBR can negate this effect.

### 2.3. Computational Modeling Predicted BBR Targets Associated with PI3K/AKT and MAKP Pathways

**Figure 3:**
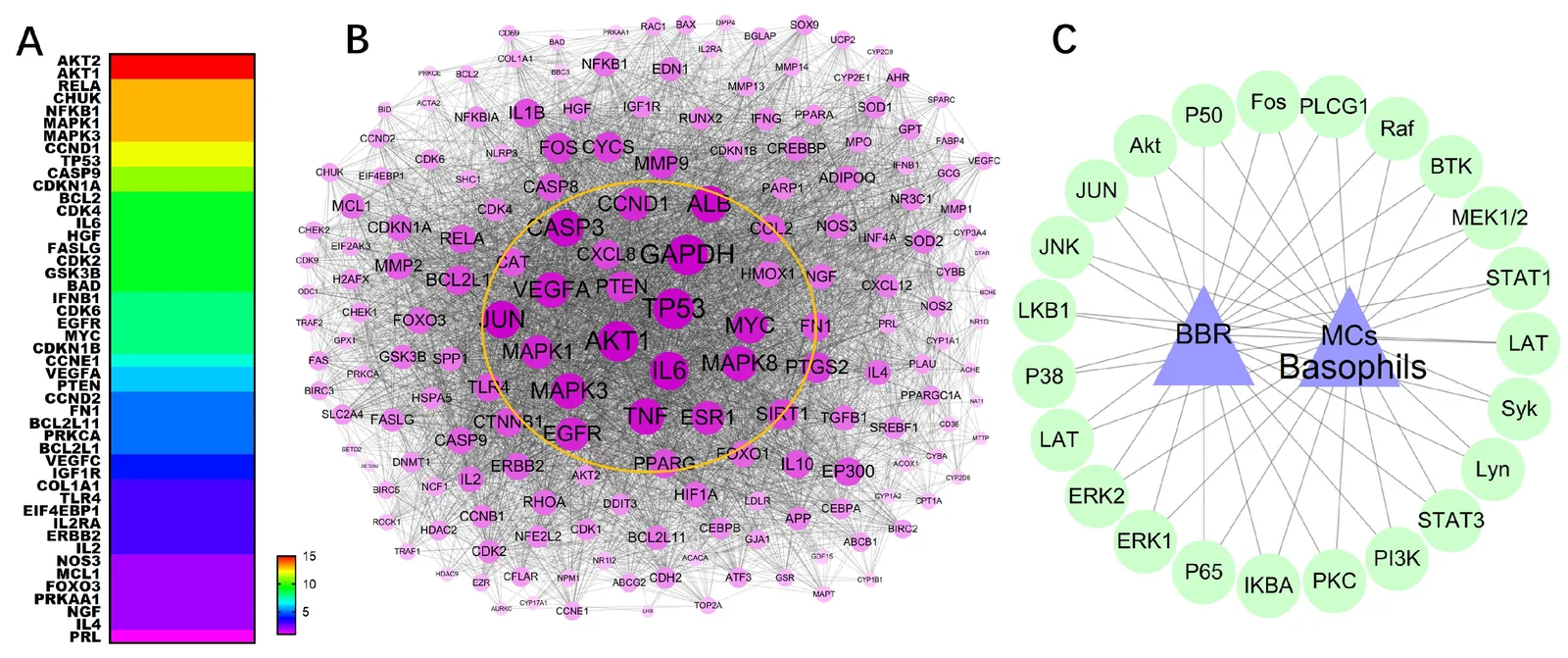
Computational modeling identified the molecular targets of BBR on MCs. (**A**) Heat map of BBR on the PI3K/AKT pathway. Biological targets of the BBR compound BBR were identified from Ensemble Approach, PubChem, and DrugBank. The relevant human genes associated with food allergy, MC, and anaphylaxis were selected as drug targets from various databases including the Therapeutic Target Database, Genetic Association, Database, GeneCards, Open Targets Platform, and Comparative Toxicogenomics Database. The shared targets of BBR, FA, MC, and anaphylaxis were obtained and mapped to KOBAS, and the PI3K/AKT pathway is the top pathway based on the frequency of genes, after excluding the cancer pathway. The heatmap of the PI3K/AKT pathway was illustrated from high degree to low degree. (**B**) Protein–protein interactions. The PPI network was constructed by mapping potential targets to the String database. The circles represent the targets. The black lines represent the interaction between nodes. Node size is proportional to its degree in the network. (**C**) Phosphorylated proteins. From the literature collection, the phosphorylated proteins associated with BBR, and MC were 89 and 61, respectively. The number of shared proteins between BBR and MC was 16 showing in green nodes; the nodes’ color indicating the direct regulated targets of berberine on MC function.

The shared targets of BBR, food allergy, mast cells, basophil and anaphylaxis were obtained and mapped to KOBAS. The PI3K/AKT pathway was found to be the top pathway based on the frequency of genes. All cancer-associated genes were excluded from the study. A heat map was created following identification of the targets. All the targets associated with PI3K/AKT pathways were categorized based on their degree from high to low. The most highly expressed targets were AKT2, and AKT1 followed by RELA, CHUK, NFKB1, MAPK1, and MAPK3 (Figure 3A). Furthermore, a protein–protein interaction (PPI) network was created by mapping potential targets to the string database. PPI showed the interaction between all these targets based on their relevance in the network. Similar to the heat map, AKT1 occupied the central position of the network. Similarly, MAPK1, MAPK3, TNF, IL6, TP53 were also highly relevant proteins, which showed high interaction in the network (Figure 3B). Next, we identified the phosphorylated proteins associated with BBR and mast cells/basophils by performing a literature search. We found 89 and 61 phosphorylated proteins associated with BBR and mast cells/basophils respectively. These proteins were mapped together, and 16 common phosphorylated proteins were found to be common between BBR and mast cell/basophil (Figure 3C). These proteins were associated with mast cell/basophil function, and it is evident that BBR has a direct effect on the functioning of these proteins. Overall, computational modeling identified key targets of BBR on mast cell/basophil functioning.

### 2.4. BBR Inhibited Standard S. aureus-Induced Expression of Genes Associated with the PI3K/AKT and MAKP Pathway

**Figure 4:**
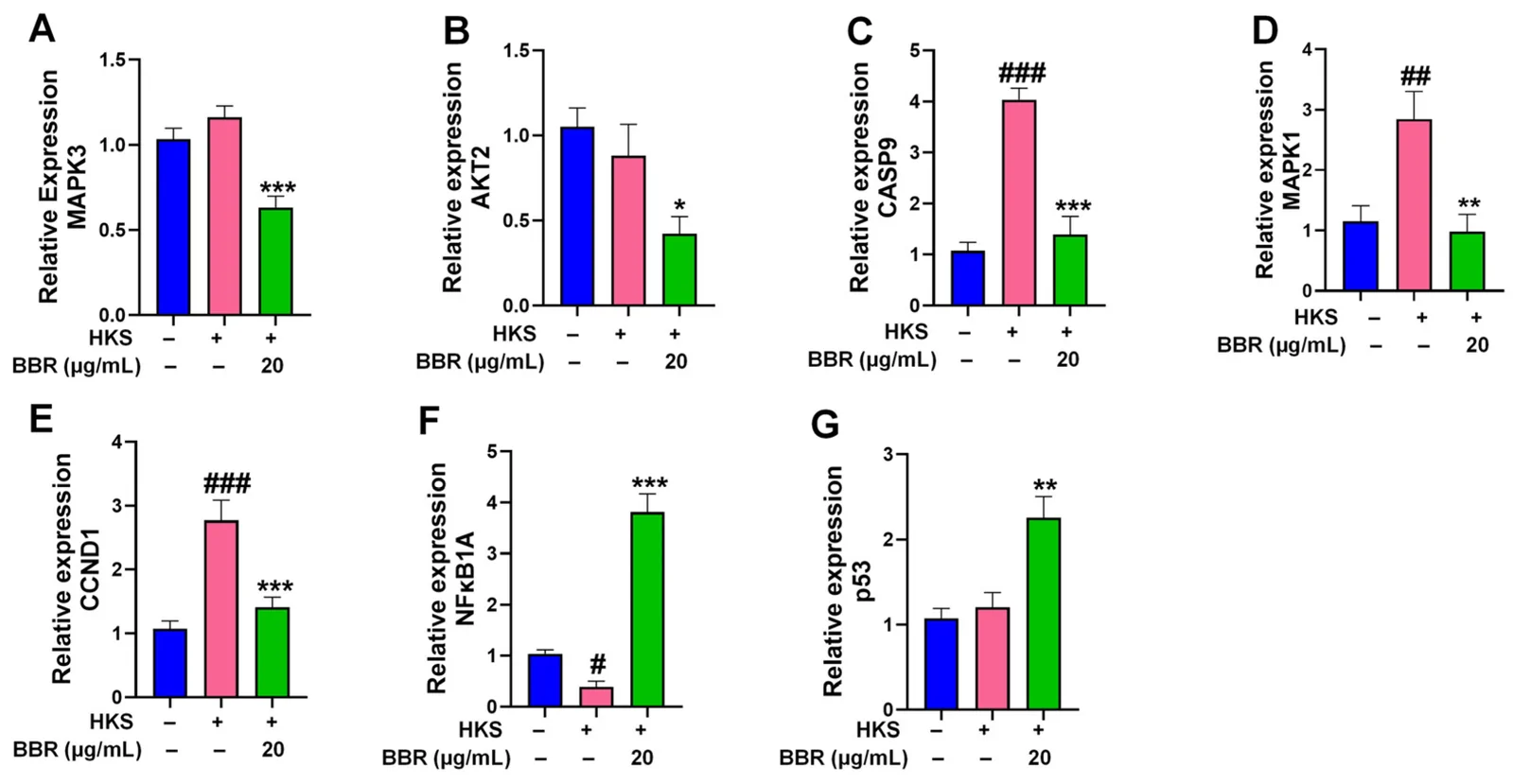
BBR showed effect on gene expressions associated with PI3K/AKT pathways. KU812 cells were cultured in presence of BBR (20 μg/mL) for 48 h. and standard heat-killed *S. aureus* (ATCC #33591) for 45 min. Cells were harvested and gene expression analysis was performed by qRT-PCR. There was a significant decrease in expression of (**A**) MAPK3, (**B**) AKT2, (**C**) CASP9, (**D**) MAPK1, (**E**) CCND1, (**F**) NfKB1A, and (**G**) p53. Blue—unstimulated/untreated; pink—stimulated/untreated; green—stimulated/treated. (N = 3 biological replicates. ^###^ *p* < 0.001; ^##^ *p* < 0.01; ^#^ *p* < 0.05 vs. untreated; *** *p* < 0.001; ** *p* < 0.01; * *p* < 0.05 vs. HKS; statistical significance was determined by one-way ANOVA). Expression was normalized using GAPDH as a housekeeping gene.

To provide experimental support and validate the highly relevant targets obtained from computational modeling, we performed a gene expression analysis. KU812 cells were stimulated with standard *S. aureus*, and expression was evaluated by qRT-PCR (Figure 4). Our results showed BBR (20 µg/mL) significantly inhibited expression of (A) MAPK3 (*p* < 0.001); (B) AKT2 (*p* < 0.05); (C) CASP9 (*p* < 0.001); (D) MAPK1 (*p* < 0.01); (E) CCND1 (*p* < 0.001). Besides PI3K activation, MAPK signaling is also involved in downstream events of basophil activation and degranulation. Therefore, inhibition of MAPK signaling is important to prevent basophil degranulation. Our results showed BBR significantly increased negative regulators of NFκB, (F) NFκB1A (*p* < 0.001), and (G) P53 (*p* < 0.001). These results show evidence that BBR has an immunomodulatory effect on transcription factors regulating basophil activation.

### 2.5. Pre-Treatment with BBR Inhibited Basophil Degranulation Following Clinical S. aureus Isolate Stimulation

**Figure 5:**
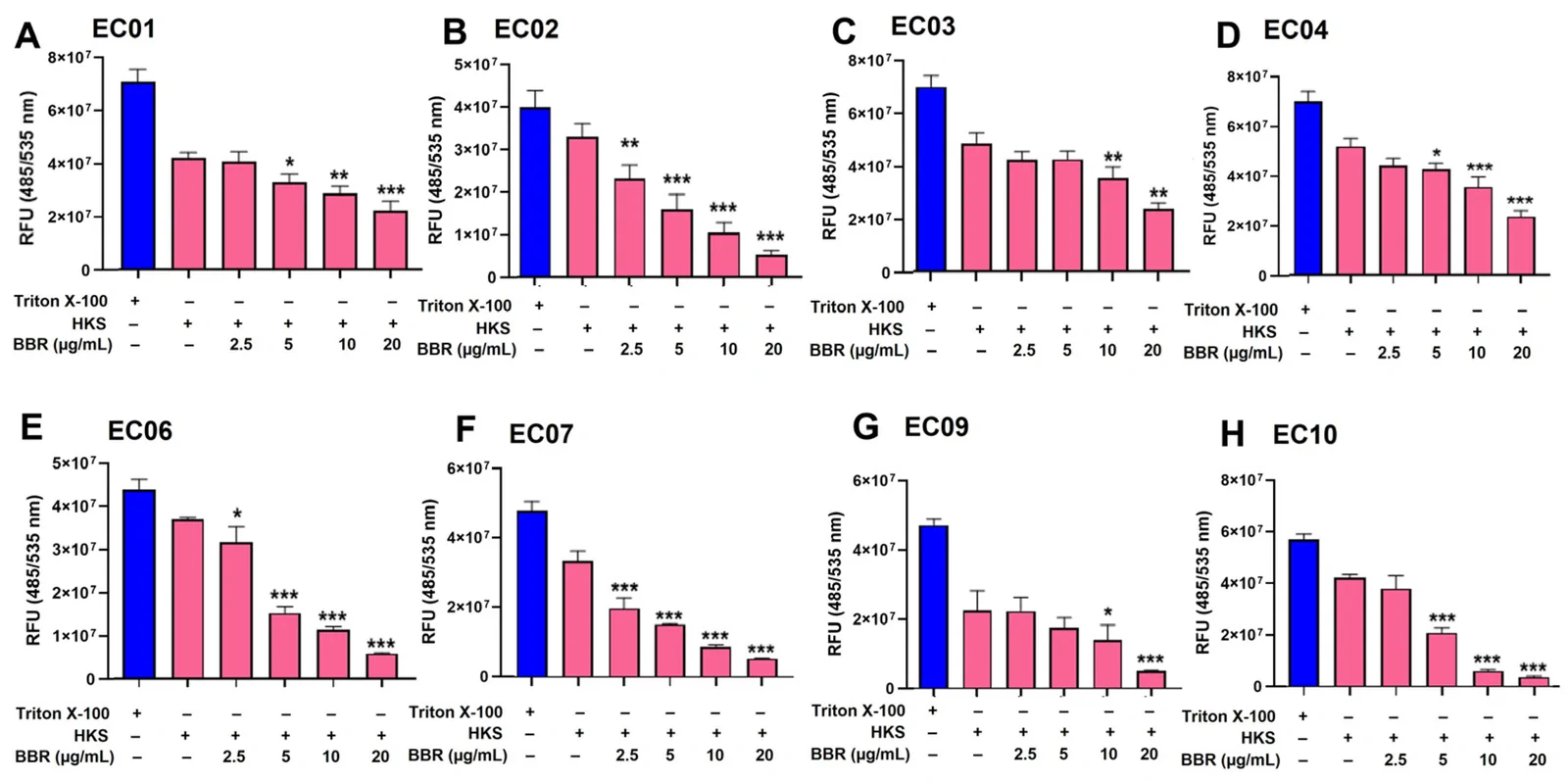
BBR inhibited β-hexosaminidase release following clinical strain *S. aureus* stimulation. Ku812 cells were pretreated with BBR at different concentrations (2.5, 5, 10, 20 μg/mL) for 48 h after which cells were stimulated with heat-inactivated *S. aureus* for 45 min. BBR treatment showed a dose-dependent inhibition of mast cell degranulation following clinical strain stimulation in (**A**) EC01, (**B**) EC02, (**C**) EC03, (**D**) EC04, (**E**) EC06, (**F**) EC07, (**G**) EC09 and (**H**) EC10. Data represents mean ± SD. Blue—unstimulated/untreated; pink—stimulated/treated (N = 3 biological replicates; *** *p* < 0.001; ** *p* < 0.01; * *p* < 0.05 vs. HKS). Degranulation is measured by mean fluorescent (RFU; statistical significance was determined by one-way ANOVA).

**Figure 6:**
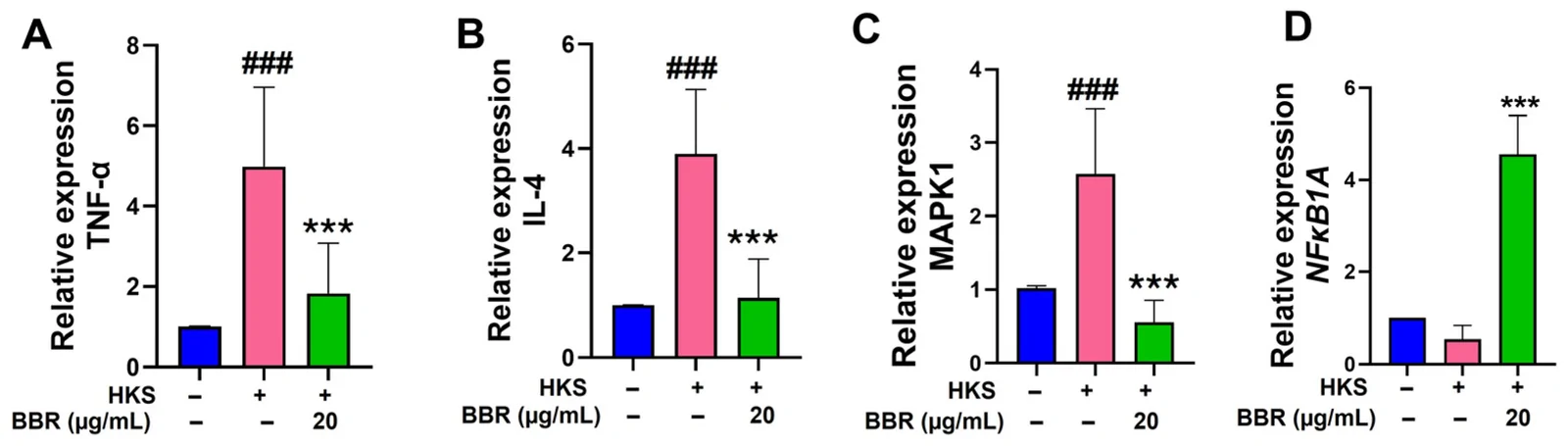
BBR modulated expression of TNF, IL-4, MAPK1 and NFκB1A following clinical *S. aureus* stimulation. Ku812 cells were cultured in presence of BBR (20 μg/mL) for 48 h. and stimulated with clinical S. aureus strain for 4 h. Cells were harvested and gene expression analysis was performed by qRT-PCR. Expression of (**A**) TNF-α, (**B**) IL-4, (**C**) MAPK1 and (**D**) NFkB1A. Blue—unstimulated/untreated; pink—stimulated/untreated; green—stimulated/treated. (N = 8 *S. aureus* clinical strains. ^###^ *p* < 0.001 vs. untreated; *** *p* < 0.001 vs. HKS; One-way ANOVA).

To study the effect of clinical *S. aureus* isolates on basophil degranulation, we stimulated KU812 cells following BBR pre-treatment for 48 h. Eight clinically isolated *S. aureus* strains were used in the study as described previously [28]. We found a dose-dependent inhibition of β-hexosaminidase release following clinical *S. aureus* stimulation. These results were consistent among all 8 clinical strains (*p* < 0.05–0.001) (Figure 5A–H). BBR (20 μg/mL) inhibited >90% degranulation in all clinical isolate stimulation. Additionally, consistent with standard *S. aureus* strain, BBR treatment significantly inhibited expression of TNF-α (*p* < 0.001) (Figure 6A) and IL-4 (*p* < 0.001) (Figure 6B) following all eight clinical strain stimulations. Overall, these results highlight BBR’s broad inhibitory effect on basophil activation following clinically different isolated *S. aureus* strains.

### 2.6. BBR Inhibited MAPK Following Clinical S. aureus Stimulation

NFκB is crucial in regulating numerous genes associated with inflammatory response. Studies suggest that MAPK can participate in the regulation of NFκB [31]. As the MAPK pathway was one of the most dominant pathways associated with BBR and mast cells/basophils based on our computational analysis, we studied the effect of BBR on MAPK1 following clinical *S. aureus* strain stimulation in KU812 cells. Our result showed significant inhibition in expression of MAPK1 (*p* < 0.001) (Figure 6C) and marked enhanced expression of NFκB1A, which is an inhibitor of NFκB (*p* < 0.001) (Figure 6D). These results are consistent with standard *S. aureus* shown previously and highlight BBR’s inhibitory effect on basophil activation via inhibiting the MAPK pathway.

## 3. Discussion

Here, we demonstrate for the first time that BBR inhibits degranulation following standard *S. aureus* ATCC (33591) stimulation in murine mast cells, rat basophils, and human basophils using the β-hexosaminidase release assay. Since the best inhibitory effect was observed in KU812 cells, we further showed BBR’s dose-dependent inhibition of β-hexosaminidase release induced by standard *S. aureus* stimulation in KU812 cells. Previous findings by Lei et al. showed increased activation of mast cells in skin abscesses induced by *S. aureus* infection [32]. Their findings highlighted the role of mast cell activation in the exacerbation of inflammation associated with bacterial infections. This study adds to the previous findings and shows *S. aureus*-mediated activation of basophils in the context of severe eczema.

The current study explored β-hexosaminidase release using a fluorometric assay. However, additional experiments including a Ca^2+^ influx assay, histamine release assay and CD63 expression need to be considered in future studies to support the claim that berberine inhibits *S. aureus*-induced mast cell/basophil degranulation. Similarly, our findings show that BBR inhibited the release of cytokines TNF-α, and IL-4 from activated basophils following standard *S. aureus* stimulation. TNF-α can contribute to inflammation, tissue damage, and immune cell activation and IL-4 enhances allergic reactions [33]. Previously, BBR’s anti-inflammatory role has been highlighted [28], and this study provides additional evidence of BBR in preventing basophil-mediated inflammation associated with *S. aureus* infection. The classical pathway of mast cell and basophil activation is via antigen/IgE [34]. FcεR1 consists of α, β and γ chains that coordinate the combined action of degranulation via the NFκB [35]. Previously, it has been shown that staphylococcal superantigen-like protein (SSL) can activate mast cell adhesion, and trigger β-hexosaminidase release [36,37]. However, it has not yet been shown how *S. aureus* contributes to basophil activation. Our results showed a significant increase in expression of FcεR subunits α, β and γ following stimulation with *S. aureus* in human basophils. This was remarkably inhibited by BBR pretreatment indicating that BBR has the potential to inhibit basophil activation induced by *S. aureus.* The exact mechanisms associated with BBR inhibition of FcεR1 (α, β and γ) induced by *S. aureus* stimulation need to be further elucidated and additional validation at the protein level should be considered in the future.

Severe AD patients typically present with substantial *S. aureus* colonization at sites of inflammatory lesions [31]. The exact quantity and specific strains of *S. aureus* present at the lesions can vary significantly from patient to patient, contributing to differences in disease severity, inflammatory responses, and mast cell/basophil activation. Recognizing the clinical relevance of strain variability [32], we isolated *S. aureus* from eight patients with severe AD to more accurately evaluate BBR as a potential therapeutic. All eight strains showed differences in genetic makeup with respect to their antibiotic-resistant genes and virulence factors. Consistent with our standard *S. aureus* results, we demonstrated that pretreatment with BBR dose-dependently inhibited β-hexosaminidase release in KU812 cells induced by stimulation with *S. aureus* isolated from severe eczema patients. Additionally, we showed inhibition of degranulation products—TNF-α and IL-4—following clinical *S. aureus* strain stimulation in Ku812 cells.

Although *S. aureus* colonizes the skin locally, it is highly likely that the toxin released from the colonized *S. aureus* circulates to the distal region through the circulation to induce degranulation. This can be validated in future studies by using specific *S. aureus*-specific toxins. These findings suggest that BBR holds significant therapeutic potential for treating *S. aureus*-exacerbated eczema through its ability to inhibit basophil degranulation. Given that *S. aureus* [11], and with the growing prevalence of multidrug-resistant *S. aureus* strains, the need for cost-effective therapies based on natural compound therapies for treatment and management becomes even more critical.

To investigate the molecular basis of BBR-mediated suppression of basophil degranulation, in silico analysis was performed to identify potential molecular targets and signaling pathways involved. Among the pathways examined, the PI3K/AKT signaling pathway was found as the most significantly associated pathway. Based on the frequency of the genes, we found that TNF-α, MAPK1, MAPK3, AKT2, CASP9, and CCND1 were highly regulated by BBR in mast cells and basophil. To provide experimental validation, we confirmed this using qRT-PCR and showed BBR significantly inhibited expression of these highly relevant genes following standard *S. aureus* stimulation in human basophil cells. In future, additional experiments need to be considered to study the effect of BBR at the protein level to better understand how it affects the phosphorylation of these targets. It has been previously shown that PI3K activation results in accelerated maturation of mast cells [38]. Multiple studies have used different strategies to modulate PI3K/Akt pathways in mast cell degranulation [22,38,39,40], as well as highlighted the role of MAPK1 encoding MAPK p38 as an important player in activation of NFκB [41,42]. P38, a member of the MAPK family, and in stimulated mast cell has been shown to be an important factor for the production of pro-inflammatory cytokines, including TNF-α [43]. Additionally, p38 MAPK contributes to regulation of different cellular functions including proliferation, pro-inflammatory cytokine production, and apoptosis [44]. To understand if this translates into human basophils, future studies need to focus on understanding how BBR influences p38 MAPK at the protein level following *S. aureus* stimulation. In addition, preclinical studies are needed to evaluate the safety, pharmacokinetics, and therapeutic efficacy of BBR to support its clinical translation in AD. To our knowledge, this is the first study to report BBR’s inhibitory effects on basophil degranulation via the changes in expression of genes associated with MAPK signaling following *S. aureus* stimulation. Similar experiments using human blood basophils would further enhance the physiological relevance of this study.

Even though the effect of BBR in *S. aureus*-stimulated human basophils seems to be promising, this study needs to be further validated using human primary mast cells such as peripheral stem cell-derived mast cells (PSCMCs) or established human mast cell lines (LAD2) to understand BBR’s role in mast cells following *S. aureus* stimulation. In addition, similar experiments using human blood basophils would further enhance the physiological relevance of this study and need to be considered in the future. Our study used a heat-inactivated *S. aureus* strain to evaluate the effect of BBR in preventing *S. aureus*-mediated mast cell/basophil degranulation under controlled, non-replicating conditions. Additional studies in future using live *S. aureus* strains will better mimic real-world interaction between *S. aureus* and mast cells/basophils. Furthermore, the present study did not access the cell viability following 48 h. of BBR treatment; however, our previous study demonstrated that 20 µg/mL BBR did not adversely affect cell viability following prolonged exposure in a different cell model [45]. Future studies should directly assess cell viability under the specific experimental conditions used here. Overall, our findings suggest that in the absence of BBR, *S. aureus* can trigger activation of basophils, leading to the release of β-hexosaminidase, TNF-α, and IL-4. In the presence of BBR, this activation is markedly attenuated, indicating a strong inhibitory effect on basophil degranulation and cytokine production. Mechanistically, it is plausible that BBR stabilizes the inhibitory IκBα, thereby preventing NF-κB translocation into the nucleus and downstream transcription of pro-inflammatory mediators. In addition, BBR suppresses AKT signaling, which may contribute to reduced IKK phosphorylation and further stabilization of the IκBα complex.

In conclusion, our findings provide compelling evidence that BBR has significant potential as a therapeutic agent in *S. aureus*-exacerbated AD. By targeting mast cell/basophil activation, inhibiting pro-inflammatory cytokine release and inhibiting MAPK-associated gene expression, BBR addresses key mechanisms driving AD pathology. Future studies are warranted to explore BBR’s efficacy and safety to support its clinical relevance.

## 4. Materials and Methods

### 4.1. Bacteria Culture and Inoculum Preparation

Standard *Staphylococcus aureus* (ATCC #33591) was purchased from ATCC (Manassas, VA, USA) and several clinical *S. aureus* strains obtained from skin from children with severe eczema undergoing topical steroid withdrawal syndrome (TSW) were coded as EC01, EC02, EC03, EC04, EC06, EC07, EC09, and EC10 respectively. First, the skin-swab sample was obtained using a sterile cotton swab and plated onto a mannitol-salt agar (MSA) plate and incubated at 37 °C for 18–22 h. to obtain isolated colonies. These isolated colonies were then streaked onto a nutrient agar (NA) and incubated at 37 °C for 18–22 h. 1 to 5 well-isolated colonies were picked from NA and subjected to sequencing to identify *S. aureus* strains. Similarly, a few colonies were re-suspended in 5 mL of nutrient broth (NB) and incubated on a shaker at 225 rpm at 37 °C for 18–22 h. The suspension was then heat inactivated by heating at 65 °C for 1 h. The heat-inactivated bacteria were re-suspended in 500 µL of sterile PBS after centrifuging at 10,000 rpm for 10 min. The concentration of bacteria was adjusted to 0.5 MacFarland standard by measuring O.D. at 600 nm using a spectrophotometer. O.D. at 0.08–0.1 was considered to be 1.5 × 10^8^ CFU/mL. Standard *S. aureus* (ATCC #33591) was also heat-inactivated following a similar procedure as described above.

### 4.2. High-Performance Liquid Chromatography (HPLC) Analysis of BBR

Berberine (BBR) used in this study was obtained from Xi’an SaiYang Biotechnology, LLC (Xi’an, China), and its composition was analyzed using high-performance liquid chromatography (HPLC) as previously described [28]. Briefly, 10 µL of BBR solution (400 µg/mL) was injected into a Waters 2690 Separation Module coupled with a 2996 photodiode array (PDA) detector (Waters, Milford, MA, USA) and separated using a ZORBAX SB-C18 column (4.6 × 150 mm, 5 µm; Agilent, Santa Clara, CA, USA). The mobile phases consisted of 0.1% formic acid in water (mobile phase A) and HPLC-grade acetonitrile containing 0.1% formic acid (mobile phase B). The flow rate was maintained at 1 mL/min. The gradient was programmed from 10% B (90% A) to 100% B over 10 min, followed by a 3 min hold at 100% B. Chromatographic data were collected at 264 nm and processed using Waters Empower 3 software. (Supplemental Figure S1).

### 4.3. Cell Culture

KU812 (human basophil progenitor cell), RBL-2H3 (rat basophilic leukemia cell), and MC/9 (a cloned mouse mast cell) lines were purchased from ATCC. KU812 cells were maintained in RPMI-1640 medium supplemented with 10% fetal bovine serum (FBS) and 1% penicillin–streptomycin, whereas RBL-2H3 and MC/9 cells were maintained in DMEM supplemented with 10% FBS and 1% penicillin–streptomycin. Culture media were replaced every 1–2 days, and cells were maintained until approximately 90% confluency was reached.

### 4.4. β-Hexosaminidase Release Assay

KU812, RBL-2H3, and MC/9 cells were cultured at 1 × 10^5^ cells in a 200 µL volume in respective culture medium in a black flat-bottom 96-well culture plate. The cells were pre-incubated with BBR at 20 µg/mL for 48 h after which cells were washed twice with 1X PBS and immediately stimulated with heat-killed *Staphylococcus aureus* (HKS) at multiplicity of infection (MOI, 2:1) for 45 min at 37 °C. The experiments comprise three different groups: 1. Cells alone; 2. Cells stimulated with HKS; and 3. Cells pre-treated with BBR followed by HKS stimulation. The β-hexosaminidase assay was performed using a β-hexosaminidase activity fluorometric assay kit (Cell Biolabs, San Diego, CA, USA). Briefly, 50 µL of each sample was transferred to a new 96-well plate and mixed with 50 µL of 1× substrate. The plate was incubated for 15 min in the dark, followed by the addition of 100 µL of 1× neutralization buffer to each well. Fluorescence was subsequently measured at excitation/emission wavelengths of 365/450 nm using a microplate fluorometer. Triton X-100 was used to induce complete cell lysis and served as the positive control.

### 4.5. Computational Analysis

Potential biological targets of BBR were identified as previously described [46]. Human genes associated with mast cells and basophils were retrieved from the Therapeutic Target Database, Genetic Association Database, GeneCards, Open Targets Platform, and Comparative Toxicogenomics Database. The top 100 genes from each database were included for further analysis.

These selected targets were mapped in Uniport Database for normalization and shared targets between BBR and mast cells and basophils were considered as potential targets for the prevention of degranulation. A compound–target–disease (C-T-P-D) network was generated using Cytoscape (v3.2.1). Identified targets were subsequently analyzed using the STRING database to determine protein–protein interactions (PPIs), and the resulting PPI network was visualized in Cytoscape (v3.2.1). 

### 4.6. Gene Expression Analysis

Expression of genes was measured by qRT-PCR. Briefly, KU812 cells were pretreated with BBR (20 μg/mL) for 48 h after which they were stimulated with HKS (ATCC#33591 and clinical strains) for 45 min. Total RNA was isolated from harvested cells using a Qiagen RNA extraction kit following the manufacturer’s protocol as previously described [28]. The purified RNA was reverse-transcribed to generate complementary DNA (cDNA). Quantitative real-time PCR (qRT-PCR) was conducted using Maxima SYBR Green/ROX qPCR Master Mix (Thermo Fisher Scientific, Millersburg, PA, USA) on a QuantStudio 5 Real-Time PCR System (Thermo Fisher Scientific, Bedford, MA, USA). Each 25 μL reaction contained 12.5 μL of 2× Maxima SYBR Green/ROX qPCR Master Mix, 1.8 μL of 0.3 μM primer assay, and 300 ng of cDNA template. Amplification was carried out for 40 cycles following the specified thermal cycling conditions. Gene expression was quantified using the comparative Ct (ΔΔCt) method, with GAPDH serving as the endogenous reference gene. Relative transcript abundance was calculated as 2−ΔΔCt and expressed as fold change relative to the corresponding control group. Primer sequences and information regarding primer design and synthesis are provided in Supplementary Table S1 and the Supplementary Methods.

### 4.7. Statistical Analysis

All statistical analyses were performed using GraphPad Prism (Version 10, San Diego, CA, USA). For all pairwise comparisons, one-way ANOVA (analysis of variance) followed by Bonferroni correction was applied for all normally distributed data. *p* values < 0.05 were considered significant. Data are expressed as Mean ± SD.

## Data Availability

Data presented in the study is included in the article; further inquiries can be directed to the corresponding authors.

## Supplementary Materials

The following supporting information can be downloaded at: https://www.mdpi.com/article/10.3390/ijms27177875/s1.

## Abbreviations

The following abbreviations are used in this manuscript:

AD	Atopic Dermatitis
MC	Mast Cell
BBR	Berberine
MAPK	Mitogen-Activated Protein Kinase
PI3K	Phosphoinositide 3-Kinase
AKT	Protein Kinase B (also known as AKT)
FcεRI	High affinity IgE receptor
IL-4	Interleukin 4
TNF-α	Tumor Necrosis Factor Alpha
TSW	Topical Steroid Withdrawal
HKS	Heat-Killed *Staphylococcus aureus*
MOI	Multiplicity of Infection
qRT-PCR	Quantitative Real-Time Polymerase Chain Reaction
GAPDH	Glyceraldehyde 3-Phosphate Dehydrogenase
PPL	Protein–Protein Interaction
HPLC	High Performance Liquid Chromatography
DMSO	Dimethyl Sulfoxide
PBS	Phosphate-Buffered Saline
SD	Standard Deviation
ANOVA	Analysis of Variance
NFκB	Nuclear Factor Kappa B
NFκBIA	NFκB Inhibitor Alpha
CASP9	Caspase9
CCND1	Cyclin D1
ATCC	American Type Culture Collection
DMEM	Dulbecco’s Modified Eagle Medium
RPMI	Roswell Park Memorial Institute medium
