# Supplementary material for: Berberine Inhibits Eczematous Skin S. aureus-Induced Mast Cell/Basophil Activation and Modulates MAPK-Associated Gene Expression
Source: Int J Mol Sci. 2026 Sep 3;27(17):7875. doi: 10.3390/ijms27177875 (PMC13566719; doi:10.3390/ijms27177875)
Supplement: Supplementary file 1 [file ijms-27-07875-s001.zip › ijms-4442638-supplementary.pdf]

Supplemental information

Supplemental Figure S1

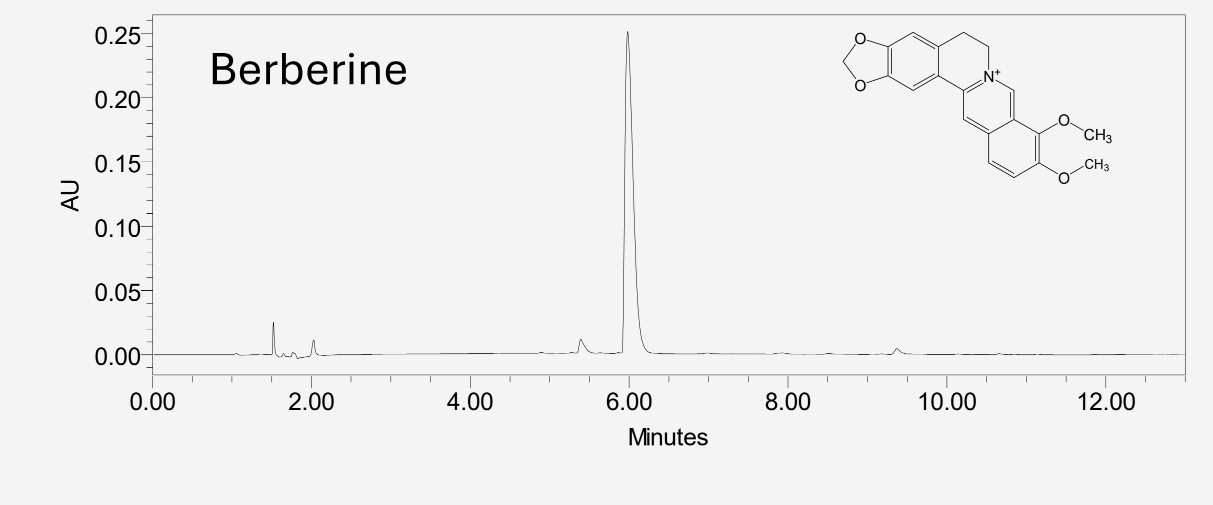

Figure S1 HPLC chromatogram and the chemical structure of berberine. BBR (40μg/mL) was analyzed on Waters 2690 HPLC system. The chromatogram was collected at wavelength of 264nm

Supplemental table S1. Primer sequence

Gene	Forward (5′-3′)	Reverse (5′-3′)
hIL-4	*CCGTAACAGACATCTTTGCTGCC*	*GAGTGTCCTTCTCATGGTGGCT*
hTNF	*CTCTTCTGCCTGCTGCACTTTG*	*ATGGGCTACAGGCTTGTCACTC*
hFcεRIα	*CTTAGGATGTGGGTTCAGAAGT*	*GACAGTGGAGAATACAAATGTCA*
hFcεRIβ;	*TAATTCTTCATAAAGACGATCATCGG*	*ATATGCCTTTGTTTTGGAACAATGGTGTG*
hFcεRIγ	*TAGGGCCAGCTGGTGTTAATGGCA*	*GATGATTCCAGCAGTGGTCTTGCT*
hMAPK1	*ACACCAACCTCTCGTACATCGG*	*TGGCAGTAGGTCTGGTGCTCAA*
hMAPK3	*AAGGGGTCAAAGGTGGAACC*	*TGCCTTGAT GACGCCGTATT*
hAKT2	*CATCCTCATGGAAGAGATCCGC*	*GAGGAAGAACCTGTGCTCCATG*

hCASP9	*GTTTGAGGACCTTCGACCAGCT*	*CAACGTACCAGGAGCCACTCTT*
hCCND	*ATGTTCGTGGCCTCTAAGATGA*	*CAGGTTCCACTTGAGCTTGTTC*
hNfKB1A	*AAG TGG TCC GCC AAG TGA AG*	*CGATTTCTGGCTGGTTAGTGATC*
hp53	*TGCGTGTGGAGTATTTGGATG*	*TGGTACAGTCAGAGCCAACCTC*

Supplemental method.

Primer design and synthesis:

Primers targeting human FcεRI β (FCER1B/MS4A2) were designed using the PrimerQuest™ primer design tool (Integrated DNA Technologies, IDT) based on the human FCER1B/MS4A2 cDNA sequence and synthesized by IDT. The human FCER1B/MS4A2 target sequence was verified against the human MS4A2 reference transcript (RefSeq NM_000139.5)."
